# Post-match recovery profile of leukocyte cell subsets among professional soccer players

**DOI:** 10.1038/s41598-021-92956-9

**Published:** 2021-06-25

**Authors:** Dorota Kostrzewa-Nowak, Paweł Wityk, Andrzej Ciechanowicz, Robert Nowak

**Affiliations:** 1 Centre for Human Structural and Functional Research, Institute of Physical Culture Sciences, University of Szczecin, 17C Narutowicza St., 70-240 Szczecin, Poland; 2 Faculty of Chemistry, Gdańsk University of Technology, Narutowicza 11/12, 80-233 Gdańsk, Poland; 3 Department of Clinical and Molecular Biochemistry, Pomeranian Medical University in Szczecin, Powstancow Wlkp. 72, 70-111 Szczecin, Poland

**Keywords:** Biochemistry, Lymphocytes

## Abstract

This study assessed the impact of cumulative match time on the distribution of CD45^+^ cell subtests in the capillary blood of professional soccer players. Twenty-two males (aged 18–30 years) took part in the 36-week study. Participants playing up to 540 in cumulative match time and less than 30 min in each single match during the observation period formed the control group. White blood cell (WBC) phenotyping and creatine kinase (CK) plasma activity analyses were performed. Also, counts for WBC subsets were determined. No significant differences in the hematological parameters or lymphocyte and NK cell percentages were observed between the control and study groups. Changes in the T cell percentage were significant during weeks 11 and 30 and in Th and Tc cell percentages during weeks 2 and 26. Significant correlations were found between the cumulative match time and Th, NK, and B cell percentages; monocyte counts; and CK activity in the control group. However, for the study group, correlations were found between cumulative match time and Th, Tc, and B cell percentages; CK activity; and the CK ratio. Our study suggests that the distribution of CD45^+^ cells might be a useful tool for monitoring the immune status of professional soccer players.

## Introduction

Intensive physical effort causes inflammation, which leads to soreness and swelling of muscle tissue. For example, the release of creatine kinase (CK) from damaged muscle cells caused by exercise or other strenuous activities is a hallmark of progressive inflammation^1–5^. A soccer match is a highly dynamic activity, and the players’ activity varies with their field position and the caliber of the teams playing the match^6^. Previously gathered data shows that the average energy spent during a match ranges between 1200 and 1700 kcal (5021–7113 kJ)^7^. Long-term and cumulative training as well as the competitive loads of the competitors often lead to an immunity disorder that is mainly characterized by decreased monocyte and macrophage percentages in peripheral blood^8–10^. Insufficient recovery time for competitors with disturbed leukocyte distribution may be one of the key factors of immunodepression^9,11,12^. One of the sport disciplines at a risk of lack of sufficient recovery time is a soccer that is characterized by multiple games with short turnarounds (~ 72 h).

Hematological studies involving physical effort usually focus on the number, size, and hemoglobin (Hb) content of red blood cells (RBCs) as well as the total hemoglobin mass (massHb)^13,14^. Since oxygen delivery to the tissues plays such an important role in exercise performance, this is a sound approach. However, white blood cells (WBCs), which protect and defend humans from infection, also play an indirect role in performance building and maintenance because ill athletes cannot participate in their training programs. WBC status data in elite athletes are often undervalued, even though they are obtained simultaneously with RBC data (from the same blood sample). Many studies contain data describing WBCs in athlete populations; however, they exclusively focus on total WBC counts^15–18^.

Although numerous immunological studies describe the molecular mechanisms of immune responses on different antigens and pathogens, relatively little is known about the factors that influence an athlete’s capacity and immune mechanisms that are responsible for the immunosuppression phenomenon induced by exercise. There are studies discussing the influence of physical exercise on salivary immunoglobulin A (s-IgA) being the first line of defense against upper respiratory tract infection in athletes^19,20^. According to them, repeated bouts of soccer-specific intermittent exercise did not influence the s-IgA concentration^19^, while playing several matches during 30-day period resulted in significant alterations in this immunoglobulin^20^. It is also known that chronic, high-intensity exercise can stimulate T cells, which results in immunosuppression^21,22^. It may manifest as increased incidences of infection, mainly in the upper respiratory system, among athletes. Previous studies demonstrate that leukocyte mobilization and functional adaptation result from acute bouts of prolonged and intense exercise^5,23–25^. Previous literature also suggests that neutrophils are mobilized in response to exercise-induced muscle damage^5,24^. The proteolytic and free radical-mediated removal of cell debris by granulocytes (mainly neutrophils) may elicit secondary tissue damage. Their phagocytic activity and communication with macrophages appear to be essential for the repair and regeneration of injured muscle tissue^26,27^. From this point of view, the first 24 h after a soccer match might be associated with a rapid immunological response followed by CD45^+^ (leukocyte) cell infiltration into damaged muscles. On the other hand, it is unclear whether leukocyte (especially lymphocyte) counts are related to the induction and execution of apoptosis and the lack of efficient hematopoietic capacity of an athlete during cumulative training loads. This is why peripheral blood mononuclear cells (PBMCs) behave differently with respect to cell death and migration during recovery time^28^. Different subsets of lymphocytes respond in different ways with respect to intensity, cell count, apoptosis markers, and cell migration markers. CD4^+^ and CD8^+^ cells appear to be prone to apoptosis after moderate exercise, but a significant increase in migration at higher intensities suggests a movement of these cells from the vasculature in post-exercise measurements^29^. Our previous study^30^ demonstrated that a post-effort increase in apoptosis among low-differentiated T cells, regarding changes in circulating T cells and progenitor cells, were found among sedentary subjects. Those changes in distribution between naïve and mature T cells seemed to be more intense in participants that train regularly^31,32^.

The immune system state of a professional athlete, especially when involved in team sports, like soccer where competition results in playing more than 30 matches each year, seems to be an important medical parameter describing the general health of the player. Literature data indicate that upper respiratory and gastrointestinal illnesses are prevalent in professional soccer^33^. Fortunately, according to the data by Orhant et al., the episodes of illness did not disrupt the trainings or matches significantly. However, those illnesses are capable of decreasing the players’ performance and ability to train hard^33^. On the other hand, it seems that CD45^+^ cell fluctuation might be a useful tool for describing recovery effectiveness based on home rest during a weekly training plan (microcycle), especially when there is more than one match per week. The purpose of this study was to evaluate the impact of cumulative match time on the distribution of leukocyte (CD45^+^) cell subtests in capillary blood among professional soccer players. To address this, professional soccer players were recruited and tested during 1 year of Polish soccer Top League competition.

## Results

**Figure 1:**
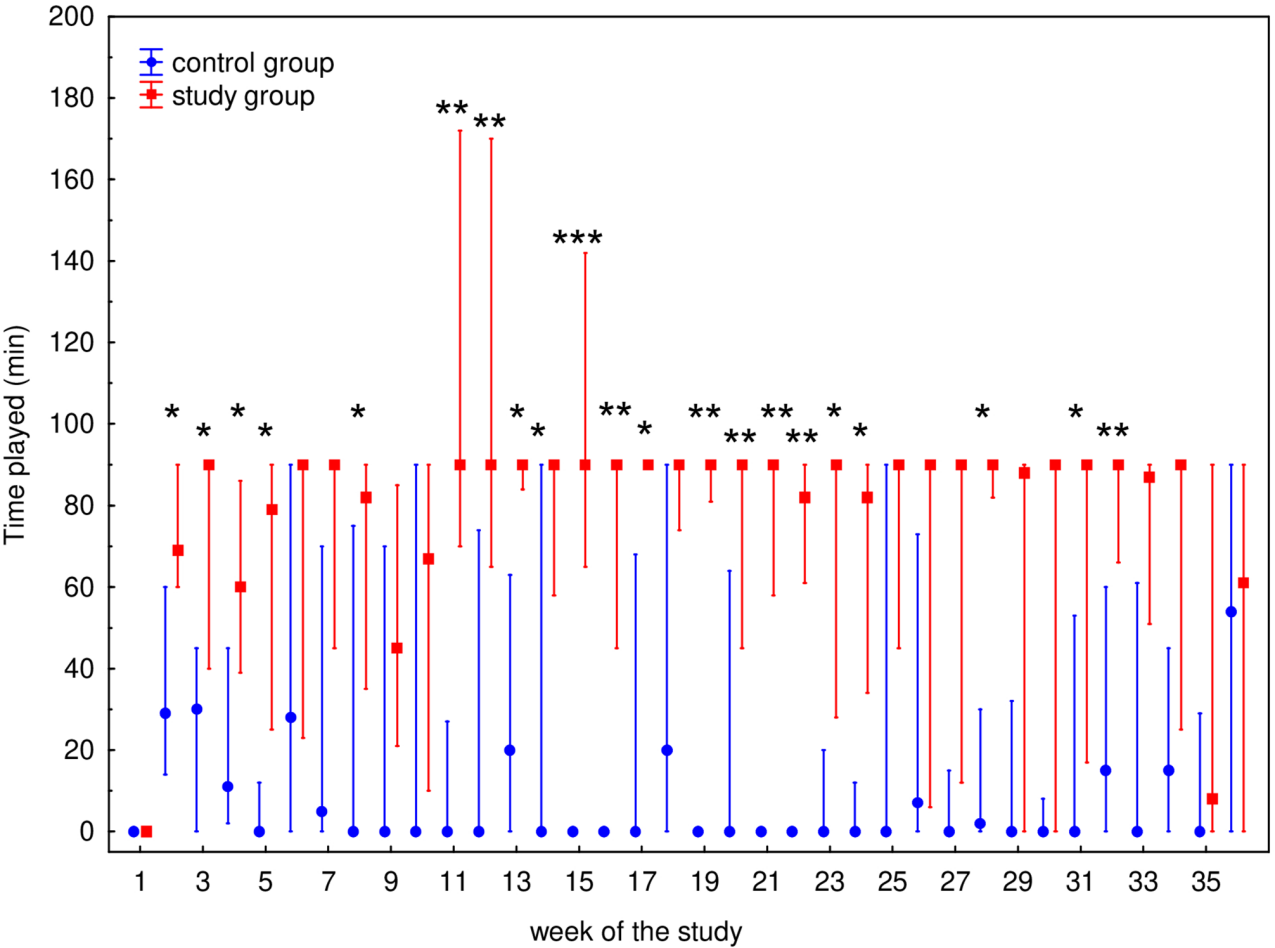
Median time played by 22 professional soccer players during 36-week study. The midpoint represents median; whiskers represent Q1–Q3 range. The significance of differences between the control and study groups was calculated using the Mann–Whitney U test. *P* value < 0.05 was considered to be significant. *P < 0.05; **P < 0.01; ***P < 0.001.

**Figure 2:**
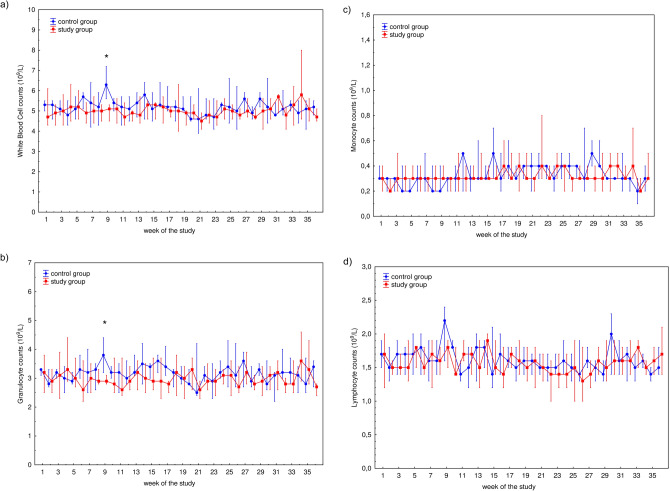
Median counts of: (**a**) white blood cells, (**b**) granulocytes, (**c**) monocytes, and (**d**) lymphocytes in studied participants’ blood measured during 36-week study among 22 professional soccer players. Blood morphology values were obtained using automatic analyzer ABX Micros 60. The midpoint represents median; whiskers represent Q1–Q3 range. The significance of differences between the control and study groups was calculated using the Mann–Whitney U test. *P* value < 0.05 was considered to be significant. *P < 0.05.

**Figure 3:**
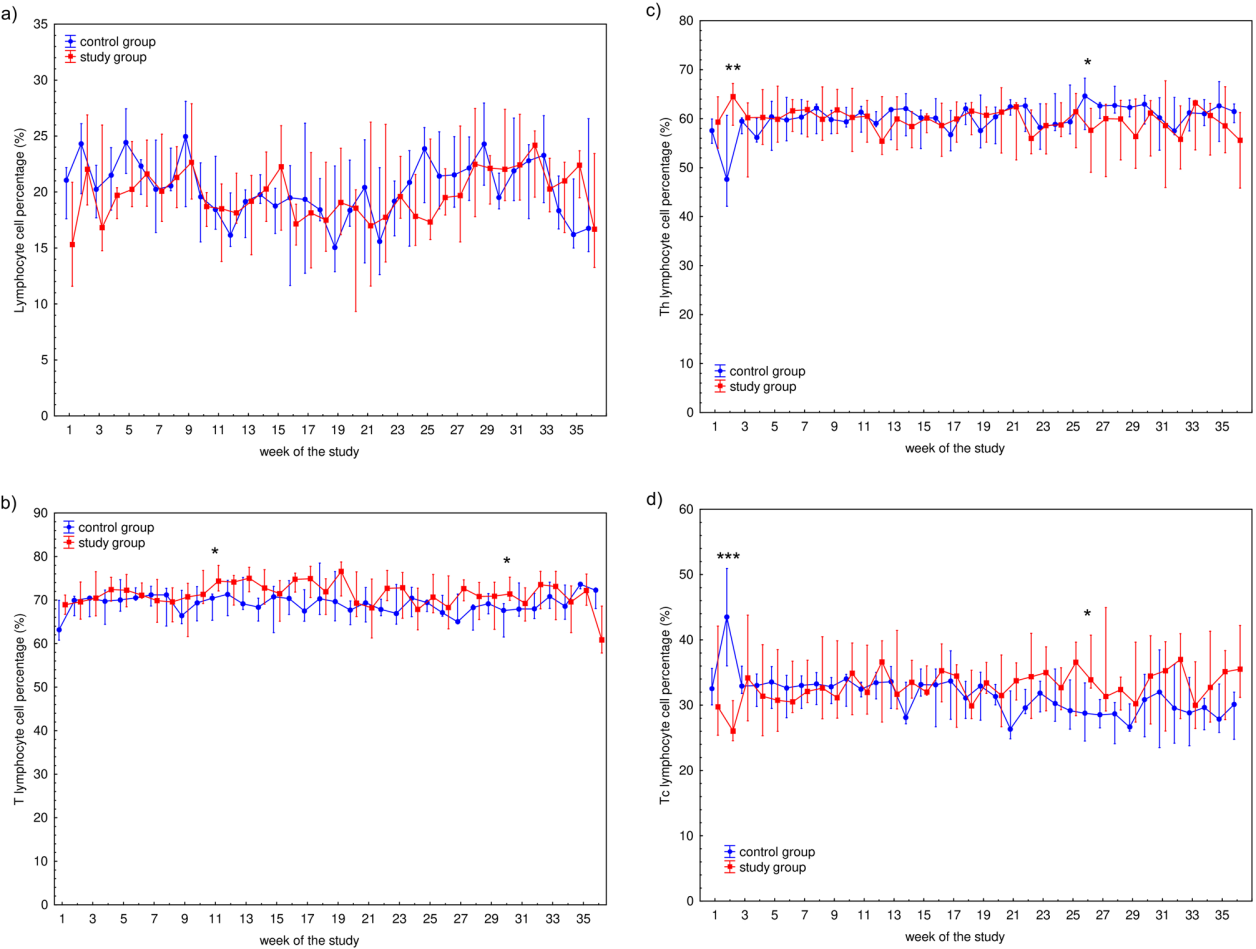

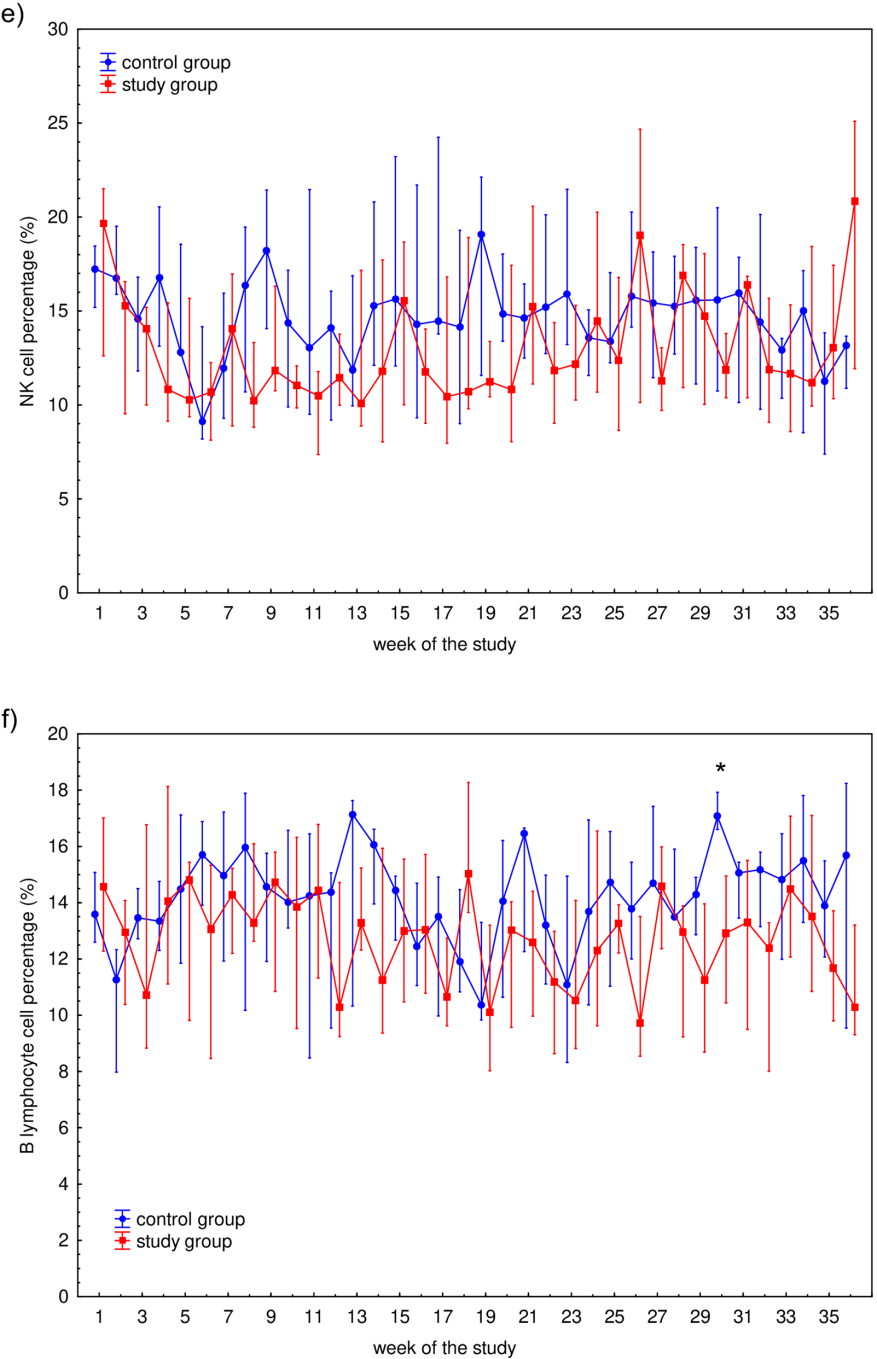
Median of the percentage of white blood cells population: (**a**) lymphocyte (CD45^+^) subsets, including: (**b**) T cells (CD3^+^), (**c**) helper/inducer T cells (Th; CD3^+^CD4^+^), (**d**) cytotoxic T cells (Tc; CD3^+^CD8^+^), (**e**) natural killer cells (NK; CD3^–^CD16^+^/CD56^+^), and (**f**) B cells (CD19^+^) of studied subjects’ blood samples measured during 36-week study among 22 professional soccer players. Blood immunophenotyping protocol was performed using commercial antibodies assay kit (BD Multitest IMK Kit) according to the manufacturer instructions and analyzed using BD Accuri C6 flow cytometer. The midpoint represents median; whiskers represent Q1-Q3 range. The significance of differences between the control and study groups was calculated using the Mann–Whitney U test. *P* value < 0.05 was considered to be significant. *P < 0.05; **P < 0.01; ***P < 0.001.

The characteristics of the participants are presented in Table 1. Median (Q1–Q3) time played was 0 (0–58) min for control group and 90 (28–90) min for study group, respectively. Mean ± SD values of time played were 26 ± 39 min for control group and 70 ± 50 min for study group, respectively. The differences in time played between these groups in each week is presented in Fig. 1. WBC counts as well as WBC subset [i.e., granulocyte (GRA), lymphocyte (LYM), and monocyte (MON)] counts during the 36-week study are presented in Fig. 2. The only significant differences in any of the hematological parameters observed between the control and study participants were WBC (*P* = 0.0434) and GRA (*P* = 0.0170) counts in week 9 (Fig. 2a,b). Fluctuations in the total lymphocyte percentages, T cells and their Th (CD4^+^), and Tc (CD8^+^) subsets, percentage of natural killer (NK) cells, and B lymphocytes are presented in Fig. 3. No significant differences were observed between the control and study groups regarding lymphocyte (Fig. 3a) and NK (Fig. 3e) cell percentages. Changes in the T cell percentage (Fig. 3b) were significant only in weeks 11 (*P* = 0.0252) and 30 (*P* = 0.0304) of the study, and differences in Th and Tc cell distribution (Fig. 3c,d, respectively) were observed only in weeks 2 (*P* = 0.0043 and *P* = 0.0005 for Th and Tc cells, respectively) and 26 (*P* = 0.0364 and *P* = 0.0364 for Th and Tc cells, respectively). Also, there was a significant difference (*P* = 0.0252) in B cell distribution during week 30 (Fig. 3f).

**Table 1:** The characteristic of the control and study groups of the participants.

Variable	Control group (*n* = 9)	Study group (*n* = 13)
Age (years)	21 (18–28)	25 (21–30)
Height (cm)	188 (175–189)	182 (179–185)
Weight (kg)	78.0 (71.0–83.0)	76.6 (72.5–80.0)
BMI (kg/m^2^)	22.7 (22.3–24.4)	22.9 (22.4–24.1)

The table presents median (Q1–Q3) values characterising the participants.

*BMI* body mass index, *n* number of participants.

Statistical analyses revealed several significant relationships between cumulative match time over 36 weeks and our variable measurements (Table 2). A moderate correlation was observed between cumulative time and Tc cell parameters in the control group (R = −0.30; *P* < 0.0001), but this correlation was trivial in the study group (R = 0.15; *P* = 0.0012). Small but significant correlations were found between Th cell percentages (R = 0.29; *P* < 0.0001), NK cell percentages (R = −0.15; *P* = 0.0085), B cell percentages (R = 0.19; *P* = 0.0007), monocyte counts (R = 0.23; *P* < 0.0001), and CK activity (R = −0.21; *P* = 0.0001) and cumulative match time in the control group. In the case of the study group, cumulative match time correlated with Th cell percentages (R = −0.18; *P* = 0.0001), Tc cell percentages (R = 0.15; *P* = 0.0012), B cell percentages (R = −0.13; *P* = 0.0035), CK activity (R = 0.24; *P* < 0.0001), and the CK ratio (R = 0.25; *P* < 0.0001) (Table 2). The CK ratio was calculated based on the CK baseline activity found in the participants at the beginning of the preparatory phase of competition (CK _(week1)_) and the CK activity measured each week during the study (CK _(week n)_). Th and B cell percentages increased with cumulative match time among the control group, but the findings in the study group were contradictory (Table 2). Interestingly, the Tc cell percentage and CK activity in the control group decreased as a function of the cumulative match time, but the opposite was observed in the study group (Table 2). Small negative correlations were found between CK activity and T or Tc cell percentages in the control group (R = −0.14; *P* = 0.0109 and R = −0.16; *P* = 0.0037 for T cells and Th cells, respectively) and between CK activity and total white blood cell (R = −0.15; *P* = 0.0008), monocyte (R = −0.17; *P* = 0.0003), and granulocyte (R = −0.13; *P* = 0.0058) counts in the study group (Table 2). Small positive correlations between CK activity and Th (R = 0.15; *P* = 0.0079) or NK (R = 0.12; *P* = 0.0270) cell percentages in the control group were found. A positive correlation between the CK ratio and Tc cell percentage distribution (R = 0.15; *P* = 0.0083) and granulocyte counts (R = 0.24; *P* < 0.0001) was found in the control group (Table 2). A small, yet significant, positive correlation was found between the CK ratio and the percentage distribution of total lymphocyte (R = 0.19; *P* < 0.0001) count, NK cell (R = 0.13; *P* = 0.0035), and lymphocyte (R = 0.22; *P* < 0.0001) percentages in the study group as well as a negative correlation between the CK ratio and the percentage distribution of B cells (R = −0.14; *P* = 0.0023) and granulocyte counts (R = −0.13; *P* = 0.0056) (Table 2).

**Table 2:** Correlation parameters between control and study group.

Parameter	Control group (*n* = 9)		Study group (*n* = 13)	
	R Spearman	P	R Spearman	*P*
**Cumulative match time (min) &**				
Lymphocytes (%)	− 0.07	0.1761	0.01	0.7595
T cells (%)	0.09	0.1132	− 0.05	0.3108
Th cells (%)	0.29	< 0.0001	− 0.18	0.0001
Tc cells (%)	− 0.30	< 0.0001	0.15	0.0012
NK cells (%)	− 0.15	0.0085	0.09	0.0541
B cells (%)	0.19	0.0007	− 0.13	0.0035
WBC (10^9^/L)	0.08	0.1542	− 0.07	0.1387
LYM (10^9^/L)	− 0.08	0.1715	− 0.11	0.0206
MON (10^9^/L)	0.23	< 0.0001	0.08	0.0827
GRA (10^9^/L)	0.083	0.1713	− 0.06	0.2120
CK (U/L)	− 0.21	0.0001	0.24	< 0.0001
Ratio CK_(week n)_/CK_(week 1)_	− 0.01	0.8176	0.25	< 0.0001
**CK (U/L) &**				
Lymphocytes (%)	− 0.04	0.4793	0.10	0.0337
T cells (%)	− 0.14	0.0109	0.06	0.2266
Th cells (%)	0.15	0.0079	− 0.05	0.2575
Tc cells (%)	− 0.16	0.0037	0.03	0.4484
NK cells (%)	0.12	0.0270	− 0.10	0.0383
B cells (%)	0.08	0.1514	− 0.07	0.1147
WBC (10^9^/L)	− 0.01	0.8843	− 0.15	0.0008
LYM (10^9^/L)	0.05	0.3639	− 0.02	0.6835
MON (10^9^/L)	− 0.01	0.7841	− 0.17	0.0003
GRA (10^9^/L)	− 0.04	0.4831	− 0.13	0.0058
**Ratio CK**_**(week n)**_**/CK**_**(week 1)**_** &**				
Lymphocytes (%)	− 0.08	0.1497	0.22	< 0.0001
T cells (%)	− 0.08	0.1536	− 0.06	0.2121
Th cells (%)	0.07	0.2370	0.02	0.6097
Tc cells (%)	0.15	0.0083	0.03	0.5143
NK cells (%)	0.07	0.1915	0.13	0.0035
B cells (%)	0.06	0.3097	− 0.14	0.0023
WBC (10^9^/L)	0.14	0.0125	− 0.04	0.3720
LYM (10^9^/L)	− 0.07	0.2384	0.19	< 0.0001
MON (10^9^/L)	− 0.03	0.5869	− 0.01	0.9256
GRA (10^9^/L)	0.24	< 0.0001	− 0.13	0.0056

The correlations between analyzed variables were assessed using Spearman’s rank correlation coefficient determination.

*BMI* body mass index, *CK* creatine kinase, *GRA* granulocytes, *LYM* lymphocytes, *MON* monocytes, *WBC* white blood cells, *n* number of participants.

## Discussion

Several studies have demonstrated that mobilization and functional adaptation of leukocytes result from acute bouts of prolonged and intense exercise^5,23–25,34,35^. The migration of leukocytes from tissues into the peripheral blood depends not only on the intensity but also on the repeatability and duration of exercise^10^. Although the clinical consequences of long-term repetitive high-intensity exercise, including soccer, are associated with immunosuppression^36,37^, such immunosuppression depends on the relative intensity of exercise as well as the appropriate exercise protocol^38^. Our previous studies showed that specialized movement causes general changes in clinical chemistry variables but did not cause an increase in CK activity during recovery^39,40^. The level of biological adaptation to physical exercise by participants is one factor related to post-effort inflammatory symptoms^39^. One of the most common post-effort observations is exertional lymphocytopenia, which usually occurs 30–60 min after the end of exercise^41–43^. If leukocytopenia, including lymphocytopenia manifested after a soccer match, the recovery period compensated for it since it was not observed in our study. Similarly, in our previous study no leukocyto- and lymphocytopenia was found 17 h after a maximal exercise bout among professional soccer players^32^ and physically active young men^44^. Although previous studies suggest that neutrophils are mobilized in response to exercise-induced muscle damage^5,24^, we did not observe significant changes in total WBC counts or in granulocyte distribution in subjects after post-match recovery. While changes in the post-match distributions of different WBC subsets (namely lymphocytes and monocytes) were observed among junior soccer players^45^, it was not confirmed during long-term observation among senior soccer players. This suggests that the adaptive mechanism and adequate recovery are important factors in immunobalance as represented by the WBC distribution in capillary blood. Such a hypothesis is related to the fact that proteolytic- and free radical-mediated removal of cell debris by granulocytes (mainly neutrophils) may elicit secondary tissue damage. Their phagocytic activity and communication with macrophages appear to be essential for the repair and regeneration of injured muscle tissue^26,27^.

Schlagheck et al. suggest investigating lymphocyte subsets to assess cellular immune responses to acute exercise and emphasize that factors such as type, duration, and intensity of exercise must be considered when analyzing the influence of exercise on cellular immune responses^34^. The role of T cells and their subsets are critical in the immune response. Th cells help facilitate inflammation as a post-effort, long-term biological effect^31,32,44^. Brown et al. indicate that T cells, and particularly their Th subset, are among the first components of the immune response activated as a biological response to physical exercise^46^. Therefore, this subset is an important tool in monitoring recovery effectiveness among professional athletes, including professional soccer players. In the present study, we did not observe any significant changes in capillary blood T cell percentage in either the study or control group after the post-match recovery time. Simpson postulated that the number of lymphocytes usually reaches a resting value in the peripheral blood up to 24 h after exercise^47^. However, our previous studies showed that the peripheral blood lymphocyte distribution in young, physically active men 17 h after progressive effort^32,44^ significantly differed from pre-exercise values. Navalta et al. also showed changes in the distribution of Th and Tc cells following 3-day, high-intensity interval exercises, which resulted from pro-apoptotic pathway activation and lymphocyte migration from lymphoid tissues to the peripheral blood^28^. From this point of view, a lack of significant changes after the recovery time might be related to the biological adaptation of the immune system to inflammation pathways involved in the post-effort immune response. It seems that the cell balance in capillary blood is disordered only in non-professional athletes and after inadequate recovery time^45^.

On the other hand, the correlation found between CK ratio and the studied cells suggests that NK cells seem to be related to post-match micro-injuries of muscle tissue. This observation is consistent with Malm et al., who suggested a close connection between muscles and blood regarding alterations in immunological variables, especially monocytes and NK cells^48,49^. Additionally, monocytes and NK cells appear to regulate immunological events in human skeletal muscles^48^. This might be associated with the rapid immunological response followed by their infiltration into damaged muscles and with the sampling time (24 h after the soccer match). The role of NK cells in recovery is consistent with other authors’ observations for non-athletes 24 h after eccentric cycling^48,50^. NK cell function is also elevated in well-trained subjects, and NK cells are the leukocyte population most influenced by physical exercise^48,51,52^. This is also consistent with our previous observation regarding junior soccer players. Taking these matters into account, we propose that NK lymphocytes are influenced by physical exercise, related to both training loads and acute exercise.

Taking the limitations of the study into account, it must be stated that the diet was not monitored although it was recommended by the club dietician to include players’ energy requirements. Also, the recovery including sleep time was not strictly monitored. The participants were asked to rest as usual with their routine, not to introduce additional variables. For the organizational reasons, blood sampling was not always at the same time after the end of the match that could have influenced the variability of the results.

### Practical applications

It is well known that temporary changes in white blood cell distribution and cell count depend on the hormonal and metabolic stress responses, which are influenced by the type of recovery^34,53^. Literature data confirms that disruption in leukocyte distribution can lead to immunosuppression^9,11^ that may lead to e.g. upper respiratory tract infections eliminating the player from training and matches. Annual competitions schedule including not only Polish Top League but also Polish Cup combined with training microcycles show high strain to the professional soccer players. On average, there is a one Top League match per week. The Cup matches are played as long as the team wins. In that situation the players may have to play at least two matches weekly. Our present study indicates that the distribution of leukocytes might be a useful tool to monitor the immune status of professional soccer players. It can enrich regular sport diagnostics focused on analyzing complete blood count with differential analysis. This approach could help coaches adjust the training process not to lead to immunosuppression of their players.

## Materials and methods

### Study design

This observational study was performed throughout the whole soccer league season (36 weeks) from July until June of the following year. The total competitive minutes of each player were registered across the whole season. The time spent on the field was the differentiating factor in the study. The participants were divided into control and study groups according to their cumulative match time. During 1 year of league and non-league competition, 45 competitive matches were played by the participants. Additionally, 6 pre-season (friendlies) matches were played during the study. The participants who did not play more than 540 min of cumulative match time and did not play more than 30 min in each single match during the observation period constituted the control group. In most cases the participants played one match per week except for few weeks when they played one Polish Top League and Polish Cup matches.

### Participants

Twenty-two male professional soccer players (median age = 24.5 years; range = 18–30 years; mean ± SD = 23.9 ± 3.7 years) with at least 8 years of training experience and belonging to the same soccer club for at least 6 months were recruited for the study. The participants had no history of metabolic syndrome (according to the International Diabetes Federation description: diabetes, prediabetes, abdominal obesity, high cholesterol, and high blood pressure) or cardiovascular disease (defined by WHO as disorders of the heart and blood vessels). Participants were non-smokers and refrained from taking any medications or supplements known to affect metabolism. No any additional types of recovery occurred during the experiment, since it was desired to analyze participants’ selected blood parameters during club’s common recovery practice. The club dietician advised diet for each participant, and the club physician monitored the participants’ health conditions.

### Methods

This study was approved by the Local Ethics Committee at the Regional Medical Chamber in Szczecin (approval no. 13/KB/V/2014). Participants were fully informed of any risks and possible discomfort associated with the experimental procedures before giving their informed written consent to participate. All methods were performed in accordance with the relevant guidelines and regulations, including the Declaration of Helsinki. Body mass of the participants was determined using a Body Composition Analyzer Tanita BC-418MA (Tanita, Tokyo, Japan). Fingertip capillary blood samples were collected according to standard diagnostic procedures^54^ 17–24 h into home recovery after match day. Capillary blood collection systems containing either lithium heparin for biochemical analyses or ethylenediaminetetraacetic acid (EDTA) for hematological and flow cytometric analyses were used.

All WBC counts and WBC subset (i.e., GRA, LYM, and MON) counts were determined within one hour after blood sampling using an ABX Micros 60 (Horiba ABX, Warsaw, Poland) hematology analyzer. The intra- and inter-assay variation was < 2.5% and < 4.0%, respectively. CK plasma activity was analyzed using an Auto Chemistry Analyzer BM-100 (BioMaxima S.A., Lublin, Poland). according to a standard diagnostic method and following the manufacturer’s instructions (BioMaxima S.A., Lublin, Poland). The intra- and inter-assay variation was < 2% and < 4%, respectively. All analyses were verified using a multiparametric control serum and control sera with normal (BioNorm) and high (BioPath) (BioMaxima S.A., Lublin, Poland) levels.

WBC phenotyping was performed using a BD Multitest IMK kit (BD Biosciences, San Jose, CA, USA) and a BD Accuri C6 flow cytometer (Becton Dickinson, Franklin Lakes, NJ, USA). The expression of surface markers was determined according to the manufacturer’s protocol. Briefly, an antibody cocktail was used to determine the percentages of T lymphocyte subsets in erythrocyte-lysed blood samples. The antibody cocktail included fluorescein isothiocyanate (FITC)-labeled CD3, phycoerythrin (PE)-labeled CD8, peridinin chlorophyll protein (PerCP)-labeled CD45, and allophycocyanin (APC)-labeled CD4. After incubating the blood samples with the appropriate aliquots of the antibody cocktail (15 min at room temperature and in darkness), a lysing solution was added. The samples were incubated in darkness (15 min at room temperature) and then analyzed by flow cytometry (BD Accuri C6, Becton Dickinson). For each sample, the fluorescence signal of at least 10^4^ gated for the forward and side light-scatter characteristics of lymphocytes was measured. The results were calculated using BD Accuri C6 (ver. 1.0.264.21) and FCS Express (ver. 4.07.0020 RUO Edition; De Novo Software, Los Angeles, CA, USA) software. The intra- and inter-assay variation was < 9.3% and < 10.1%, respectively.

All samples were analyzed be the same experienced researcher to minimize as much as possible the influence of inter-assay variation.

### Statistical analyses

All data are presented as the median (Q1–Q3). Statistical analyses were performed using STATISTICA version 13 software (2017; TIBCO Software Inc., Palo Alto, CA, USA). The normality of the data distribution was analyzed using the Shapiro–Wilk W test. The significance of differences between analyzed time points (consecutive observation weeks of soccer league) was calculated using Friedman’s analysis of variance for repeated measures followed by a post-hoc Dunn’s test with Bonferroni correction. The significance of differences between the control and study groups was calculated using the Mann–Whitney U test. The correlations between analyzed variables were assessed using Spearman’s rank correlation coefficient determination. For each analysis, a *P* value < 0.05 was considered to be significant.

## Data Availability

The datasets generated and/or analyzed during the current study are available from the corresponding author on reasonable request.
